# Ultrasound-guided transversus thoracic muscle plane-pectoral nerve block for postoperative analgesia after modified radical mastectomy: a comparison with the thoracic paravertebral nerve block

**DOI:** 10.1186/s13741-022-00270-3

**Published:** 2022-07-27

**Authors:** Ying Zhao, Weilin Jin, Peng Pan, Shuquan Feng, Danyun Fu, Junyan Yao

**Affiliations:** 1 Department of Anesthesiology, Kunshan Hospital of Traditional Chinese Medicine, No.189, Chaoyang road, Yushan District, Kunshan, 215300 Jiangsu China; 2 Department of Anesthesiology, Shanghai General Hospital, Shanghai Jiao Tong University School of Medicine, No.100, Haining Road, Hong Kou District, Shanghai, 200080 China

**Keywords:** Analgesia, TTP-PECS, TPVB, Modified radical mastectomy, Early recovery

## Abstract

**Background:**

Modified radical mastectomy (MRM) is the most effective and common type of invasive surgery for breast cancer. However, it causes moderate to severe acute pain and even lasts for a long postoperative period. Transversus thoracic muscle plane-pectoral nerve block (TTP-PECS) is a novel and promising interfacial plane block which can provide analgesia for MRM while thoracic paravertebral nerve block (TPVB) is also widely used for this purpose. This study compared the postoperative analgesia between the ultrasound-guided TTP-PECS and TPVB in patients undergoing MRM.

**Methods:**

In this randomized controlled trial, eighty female breast cancer patients undergoing unilateral MRM with sentinel lymph node dissection (SLND) and axillary dissection (ALND) were enrolled. Patients were randomized to receive either ultrasound-guided TTP-PECS (TTP-PECS group, *n* = 40) or TPVB (TPVB group, *n* = 40) with 0.5% ropivacaine 30 ml. Evaluated variables included 24 h postoperative total PCA fentanyl consumption, including PCA background consumption and PCA press consumption (per bolus dosage multiply by the effective pressing times), and intraoperative fentanyl consumption, as well as postoperative flurbiprofen axetil requirement, duration of analgesia, blocking area, pain intensity at rest and during activity, ability to reduce the inflammatory response, and the quality of recovery 40 (QoR-40) score of patients.

**Results:**

Compared with the TPVB, the main blocking area was *T*_2_–*T*_6_ in the TTP-PECS group, which was more suitable for the MRM. TTP-PECS has a longer analgesia duration than TPVB; 24 h postoperative total PCA fentanyl consumption, especially the PCA press consumption, and the postoperative flurbiprofen axetil requirement were decreased in the TTP-PECS group than those in the TPVB group. Furthermore, the VAS scores at rest and during activity and inflammatory response were lower in the TTP-PECS group compared with the TPVB group at 12 h postoperatively. Finally, the total QoR-40 score, especially for the scores of pain; emotional state; and patient support were better in the TTP-PECS group.

**Conclusion:**

Compared with the TPVB, TTP-PECS can provide better postoperative analgesia in patients undergoing MRM, simultaneously reduce the inflammatory response, and prompt early recovery. These results suggest that TTP-PECS is an attractive alternative to TPVB for postoperative analgesia of modified radical mastectomy.

## Background

Breast cancer is the most common malignancy in females, with an increasing incidence in recent years (Greaney et al. 2015). Surgery is one of the mainstays of therapy for breast cancer, and modified radical mastectomy (MRM) is the most effective and common type of invasive surgical treatment. Despite conventional analgesia strategies, patients still suffer from moderate to severe acute postoperative pain that can impede their early recovery (Oscar et al. 2017). Therefore, better pain management is necessary and urgently needed.

Many types of regional anesthesia techniques have been used during anesthesia for MRM (Dai et al. 2015). Thoracic epidural, intercostal nerve, and interscalene brachial plexus blocks are limited by the complicated nature of their procedures and severe complications. Based on the application of ultrasound (US), TPVB block has been used for anesthesia and has gained better abirritation during MRM. However, this technique is also limited by the complicated operation and severe postoperative complications such as hypotension, epidural or intrathecal spread, and pleural puncture (Li et al., 2016).

In recent years, a novel and less invasive regional analgesia technique known as “TTP-PECS” has received increasing interest for application in breast surgery. Two randomized clinical trials (Liang et al. 2019; Wang and Zhao, 2021) reported effective postoperative pain management using TTP-PECS than TPVB in patients undergoing MRM. However, clinical evidence largely still need further confirmation.

In the present study, our primary aim was to compare the effects of ultrasound-guided TTP-PESC and TPVB on 24 h postoperative total PCA fentanyl consumption (including PCA background consumption and PCA press consumption) after MRM. Our secondary aim was to compare intraoperative fentanyl consumption and postoperative flurbiprofen axetil requirement, duration of analgesia, blocking area, postoperative pain intensity at rest and during activity, and inflammatory response (proinflammatory cytokines including IL-6, MCP-1, and TNF-α, and pain-related mediators including PGE_2_, NPY, and β-endorphin), as well as QoR-40 score between the TTP-PESC and TPVB groups.

## Methods

### Study design

This retrospective randomized study was reviewed and approved by the Medical Ethics Committee of the Kunshan Hospital of Traditional Chinese Medicine on April 9, 2019 (Approval ID: 2019-18), and written informed consent was obtained from each patient for participation in the study. The trial was conducted from January 2017 to December 2019. The trial was registered in the Chinese Clinical Trials Registry (retrospectively registered: ChiCTR2000033943). All eligible patients were approached for enrollment.

### Eligibility criteria

Eligible studies were required to meet all the following criteria:

Studies focusing on female patients with adult breast cancer who underwent surgery. All patients were undergoing elective unilateral MRM (including SLND and ALND) with no ethnicity or nationality restrictions, age from 28 to 74 years, body mass index (BMI) of 17 to 29.9 kg/m^2^, and American Society of Anesthesiologists (ASA) status I or II.

Studies were considered to be ineligible and were excluded if they met the following criteria:

1. Patients who underwent secondary or nonradical surgery and breast reconstruction
2. Patients who had a history of infection around the puncture site, allergy, or contraindication to local anesthetics
3. Patients currently on anticoagulant treatment, alcohol or substance abuse, opioid dependence, or regularly receiving corticosteroids
4. Patients who had systemic infectious diseases or psychiatric or neurological diseases
5. Patients who did not cooperate during the procedure and the follow-up survey

After applying the inclusion and exclusion criteria, a total of 80 patients were enrolled. The randomization schedule generated a randomized list of numbers that were enclosed in sealed envelopes by a third party not involved in the study. These patients were randomly allocated to either TTP-PECS or TPVB group (*n* = 40 each).

### Preparation before anesthesia

After intravenous access was established, the patients were routinely monitored for various parameters, including heart rate, arterial pressure, pulse oxygen saturation, electrocardiography, end-tidal CO_2_, and bispectral index during the operation. All patients were administered midazolam 0.05 mg/kg prior to nerve block.

Ultrasound (US)-guided nerve blocks in each technique were performed by two anesthesiologists with more than 3 years of experience in US-guided regional anesthesia and with a record of performing more than 150 blocks. All US scans were performed using the same US machine (GE Healthcare, LOGIQ e) and a linear array probe (6 to 13 MHz frequency). The US image was optimized by adjusting parameters, including depth, penetration frequency range, and gain. The blocks were performed using a 21-gauge echogenic needle (UniPlex NanoLine 21G × 100 mm). After sterile preparation, the gel was applied to the US transducer. The transducer and cable were covered with a sterile plastic sleeve, and the skin was infiltrated with 1% lidocaine.

## Ultrasonography

### Patients in the TTP-PECS group underwent ultrasonography in the forearm outreach position

#### PECS I block (Blanco 2011)

**Fig. 1:**
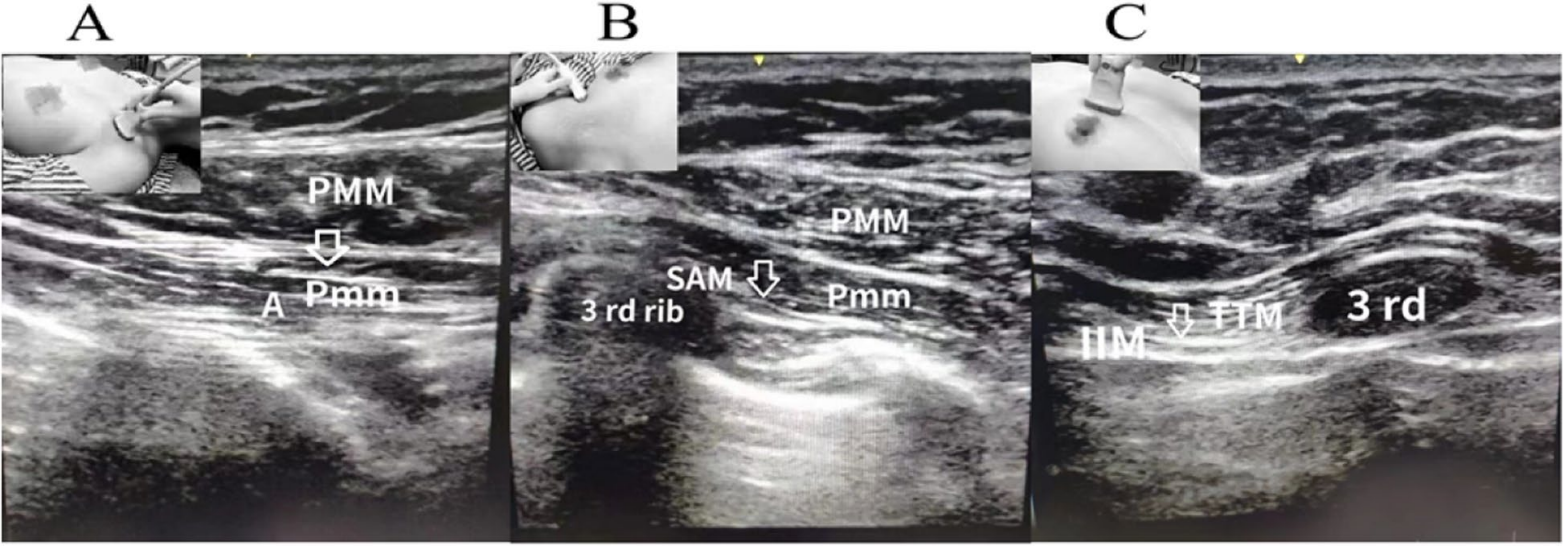
For the TTP-PECS blocks, the position of the ultrasound transducer is shown in the upper left of the images. During ultrasound scanning of PECS I block, a local anesthetic was injected in the plane between the PMM and Pmm (**A**); in PECS II block, a local anesthetic was injected in the plane between the Pmm and SM (**B**); and in TTP block, a local anesthetic was injected in the plane between the IIM and TTM (**C**). The arrow indicates the injection point. A, artery; PMM, pectoralis major muscle; Pmm, pectoralis minor muscle; SAM, serratus anterior muscle; IIM, internal intercostal muscle; TTM, transversus thoracic muscle

The US transducer was placed in the lateral third of the clavicle, where the pectoralis major and pectoralis minor muscles were easily identified. The anesthetist then confirmed the location of the pectoral branch of the thoracoacromial artery between the pectoralis muscles with color Doppler. The pectoral nerve was consistently located adjacent to the artery. The needle was then inserted in-plane of the ultrasound transducer, and 7.5 ml of 0.5% ropivacaine was injected between the pectoralis muscles (Fig. 1A).

#### PECS II block (Blanco et al. 2012)

The US transducer was moved inferolaterally until the serratus anterior muscle was identified above the 2nd, 3rd, and 4th ribs. The needle was advanced in a mediolateral direction in-plane of the ultrasound transducer, and 15 mL of 0.5% ropivacaine was injected into the fascial plane between the pectoralis minor muscle and the serratus anterior muscle (Fig. 1B).

#### TTP block (Ueshima and Kitamura, 2015)

The US transducer was finally placed in the longitudinal plane 1 cm lateral to the sternal border, where the *T*_3–4_ intercostal space was identified under US. A parasternal sagittal view of the internal intercostal muscle and the transversus thoracic muscle between the 3rd and the 4th ribs was visualized above the pleura. The needle was inserted in-plane to the transducer until the tip was located between the internal intercostal muscle and the transversus thoracis muscle, and 7.5 ml of 0.5% ropivacaine was then injected (Fig. 1C).

### Patients in the TPVB group underwent ultrasonography in the lateral position

#### TPVB block (Marhofer et al. 2010)

**Fig. 2:**
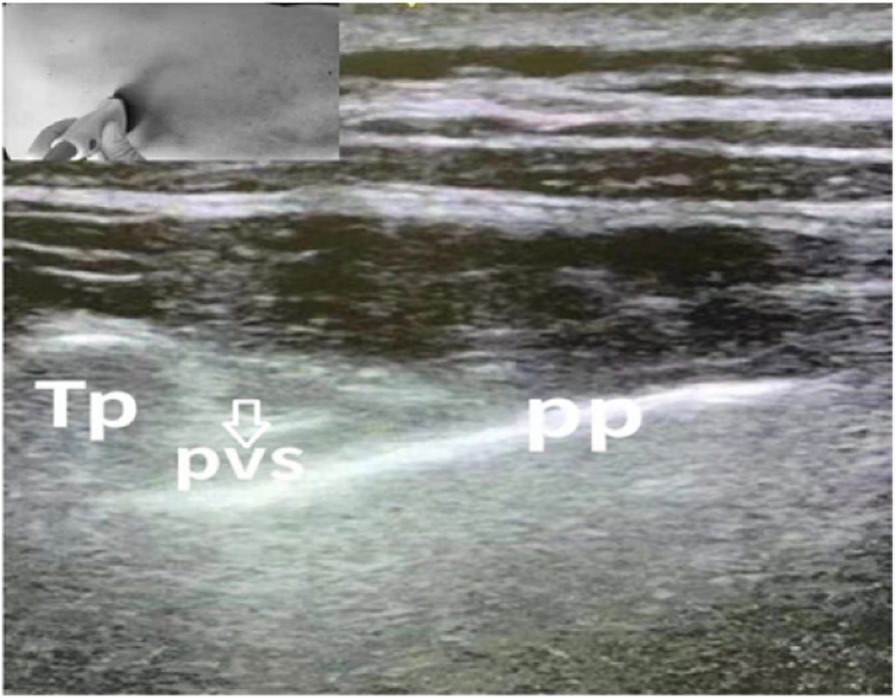
For the TPVB block, the position of the ultrasound transducer is shown as an inset in the upper left of the image. During ultrasound scanning of the TPVB block, a local anesthetic was injected into the paravertebral space. The arrow indicates the injection point. pp, parietal pleura; pvs, paravertebral space; Tp, transverse process

The US transducer was placed at the level of the 5th thoracic vertebra, in contact with the transverse process of the 6th thoracic vertebra. The needle was then passed caudally for 1–1.5 cm into the paravertebral space, and 15 ml of 0.5% ropivacaine was injected under real-time US guidance (Fig. 2). The same procedure was repeated for the 3rd thoracic vertebra.

The TTP-PECS blocks and TPVB block interventions were performed 30 min before the surgical incision. After the nerve blocks were completed, the sensory level was tested with a pin prick every 10 min by another anesthesiologist not involved in the operation. Successful block performance was defined as dysesthesia of the skin or absence of pain in any segment from T_1_ to T_12_ within 30 min after blocks. Simultaneously, any adverse effects related to the regional anesthetic technique were also recorded.

## Intraoperative management

General anesthesia was induced with 1.2 μg/kg fentanyl, 3 mg/kg propofol, and 0.2 mg/kg atracurium *cis*-benzenesulfonate for the insertion of a laryngeal mask. Initial respiratory parameters were set as follows: volume-controlled ventilator; expiratory tidal volume, 8 to 10 ml/kg; respiratory rate, 10 to 12 times/min; inhale:exhale ratio, 1:2; fraction of inspiration O_2_, 100%; and end-tidal carbon dioxide pressure, 35 to 45 mmHg. Sevoflurane was continuously infused to maintain anesthesia, and 0.05 mg fentanyl was injected as a supplemental analgesic and repeated if necessary. A bispectral monitor was used to determine the appropriate depth of anesthesia, with bispectral index values between 40 and 60.

A vasoactive drug was administered intravenously when the mean arterial blood pressure or the heart rate was reduced by 20% as compared to the preoperative baseline values.

## Postoperative management

After recovery from anesthesia, the patients were transferred to the post-anesthesia care unit (PACU). Antiemetic therapy comprised a dose of 5 mg/day prophylactic tropisetron. Postoperative analgesia was provided by intravenously patient-controlled analgesia (PCA) using fentanyl. The PCA pump contains 16μg/kg fentanyl diluted to 100 ml with normal saline, setting as 2 ml/h background infusion, demand bolus 2ml, and a lockout interval of 15 min.

Pain intensity was assessed using the VAS score at rest and during abduction of the ipsilateral upper limb at 2, 6, 12, and 24 h postoperatively. The severity of pain was classified as mild (VAS 0–3), moderate (VAS 4–7), and severe (VAS 8–10). Breakthrough pain was defined as a VAS score of 3 or more at rest or on patient’s demand: one or two PCA button themselves as the first rescue analgesic. If the VAS score was persistently 3 or more after 30 min of the first rescue analgesia, IV flurbiprofen axetil 50 mg was administered slowly as the second rescue analgesia, and the cumulative dose was less than 200 mg per day. Dynamic pain was defined as the difference in VAS score between rest and activity of > 2 points. Total fentanyl consumption in 24 h postoperatively, including PCA background consumption and PCA press consumption, and the effective pressing times of PCA pump, as well as second rescue analgesics, were recorded.

Blood samples were collected for examinations of proinflammatory cytokines including IL-6, MCP-1, and TNF-α by enzyme-linked immunosorbent assay (ELISA) and pain-related mediators including NPY, PGE2, and β-endorphin by radioimmunoassay before surgery, immediately after surgery, 12 and 24 h after surgery.

The QoR-40 score was administered 24 h after surgery. The QoR-40 Questionnaire consists of 40 items and five subscales that are divided into separate sections which aimed to evaluate the presence and extent of pain, symptoms, comfort, emotional well-being, physical independence, and satisfaction with treatment. All these items are rated on a 5-point Likert scale from 1 (worst) to 5 (best). The total score was computed by summing all items. The possible minimum and maximum scores were 40 and 200, respectively.

## Sample size calculation

By using the SPSS version 22.0 software (SPSS Inc., Armonk, NY, USA), postoperative opioid consumption in the first 24 h after surgery was considered the primary efficacy variable. Based on the results of a pre-established analysis plan for the TTP-PECS and TPVB groups (with 10 patients in each group), a mean fentanyl consumption of 525 and 625 μg, respectively (standard deviation of 150 μg), was used. The calculated sample size was 37 individuals in each group (*α* = 0.05; power = 0.8). Considering possible drop-outs, we decided to enroll at least 40 patients per group.

## Statistical analysis

The SPSS version 22.0 software (SPSS Inc.) was used for all statistical tests. Parametric data were expressed as mean ± SD, and nonparametric data were expressed as median and range. An independent Student *t*-test was used to compare the continuous data between the two groups. Categorical data were assessed using the *chi-square* test. *P* value < 0.05 was considered statistically significant.

## Results

### Experimental process

**Fig. 3:**
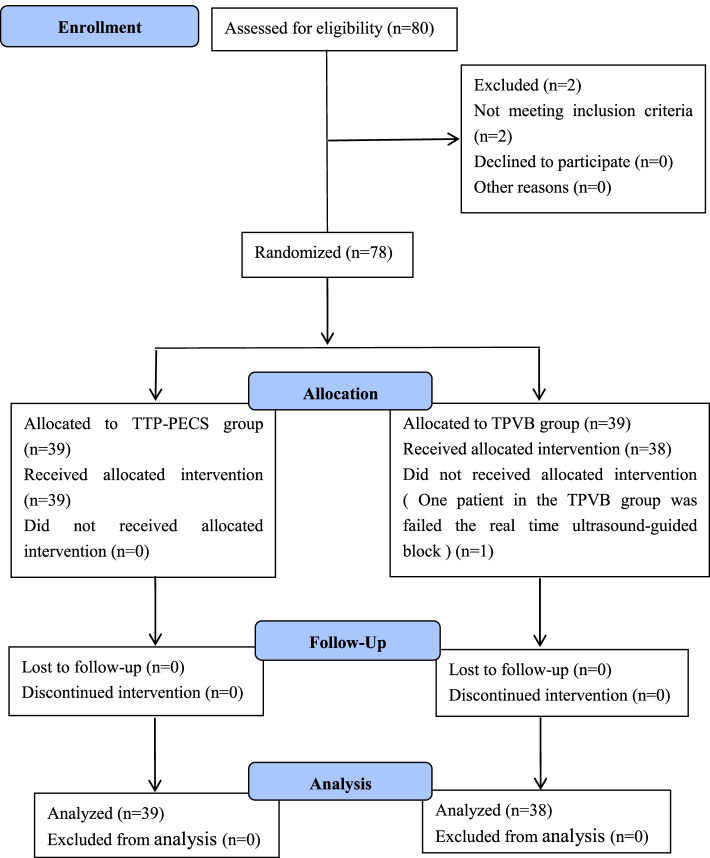
Flow diagram

Eighty patients were screened for enrollment in the present study. After applying the exclusion criteria, 78 patients were included in the randomization process (39 patients in each group). Real-time ultrasound-guided regional block was performed in all patients, but one failed in the TPVB group. Consequently, 77 patients were ultimately analyzed. Figure 3 shows the CONSORT diagram for recruitment to the trial.

### Demographic data and perioperative characteristics

The patients in the two groups were well matched for demographic data and perioperative characteristics, and no significant difference was observed between the two groups (*P* > 0.05). The details are provided in Table 1.

**Table 1:** Demographic and perioperative characteristics

Variables	TTP-PECS group	TPVB group	*P* value
Age (years)	50.87 ± 12.52	49.95 ± 11.95	**0.74**
Height (cm)	161.67 ± 4.98	161.50 ± 5.11	**0.89**
Body weight (kg)	59.00 ± 6.22	59.16 ± 6.26	**0.91**
BMI (kg/m^2^)	22.68 ± 3.15	22.78 ± 3.10	**0.89**
ASA grade (*n*, I/II)	13/26	11/27	**0.68**
Duration of operation (min)	115.20 ± 18.88	115.29 ± 18.54	**0.98**
Total loss of blood (ml)	70.51 ± 17.46	71.84 ± 16.58	**0.73**
Infusion volume (ml)	997.44 ± 126.67	986.84 ± 114.30	**0.70**

Demographic and perioperative characteristics were taken before and during the surgery. No significant differences were observed in age, body weight, height, BMI, ASA grade, duration of operation, total loss of blood, and infusion volume among groups (*P >* 0.05). Data are expressed as mean ± SD (39 in the TTP-PECS group and 38 in the TPVB group)

*BMI* body mass index, *ASA* American Society of Anesthesiologists

### Fentanyl consumption, flurbiprofen axetil requirement, and the duration of analgesia

Although intraoperative fentanyl requirement and PCA background consumption were similar between the two groups (*P >* 0.05), compared with the TPVB group, the duration of analgesia was longer in the TTP-PECS group (*P* < 0.01); 24 h postoperative total PCA fentanyl consumption, especially the PCA press consumption; the effective pressing times of the PCA pump; and the rate of postoperative flurbiprofen axetil requirement were all decreased in the TTP-PECS group (*P* < 0.05), as shown in Table 2.

**Table 2:** Fentanyl and flurbiprofen axetil consumption and the duration of analgesia

Variables	TTP-PECS group	TPVB group	*P* value
Intraoperative fentanyl consumption (μg)	278.1 ± 42.0	257.9 ± 50.0	**0.06**
Duration of analgesia (h)	12.5 ± 1.3*	9.4 ± 1.7	**0.00**
24 h postoperative total fentanyl consumption (μg)	547.33 ± 57.79*	701.02 ± 93.71	**0.00**
PCA background consumption (μg)	453.12 ± 47.77	454.33 ± 48.10	**0.91**
PCA press consumption (μg)	94.21 ± 33.60*	246.69 ± 57.71	**0.00**
The effective pressing times of PCA pump (time)	5**.**05 ± 1.88*	13.00 ± 2.53	**0.00**
Postoperative flurbiprofen axetil requirement (*n*/*n*) (%)	1/38 (2.6)*	8/30 (26.7)	**0.03**

Fentanyl and flurbiprofen axetil consumption and the duration of analgesia were recorded in the first postoperative 24 h. TTP-PECS treatment significantly reduced the 24 h postoperative total fentanyl consumption, especially the PCA press consumption, the effective pressing times of PCA pump, and the rate of patients requiring flurbiprofen axetil, and prolonged the duration of analgesia. Data are expressed as mean ± SD (39 in the TTP-PECS group and 38 in the TPVB group)

*P < 0.01 or 0.05, versus the TPVB group

### Area blocking

The main blocking area was *T*_2~6_ and the axillary region in the TTP-PECS group, but was *T*_2~7_ in the TPVB group; only 18 cases reached the axillary region (*P* < 0.01), as shown in Table 3. Axillary region dermatomal spread was significantly increased, but *T*_6_ and *T*_7_ dermatomal spread decreased in the TTP-PECS group, which was more suitable for the MRM.

**Table 3:** Area blocking between the two groups

	Axillary region	*T*_2_	*T*_3_	*T*_4_	*T*_5_	*T*_6_	*T*_7_
TTP-PECS group	37 (94.9)*	39 (100)	39 (100)	39 (100)	38 (97.4)	30 (76.9)*	14 (35.9)*
TPVB group	18 (47.4)	18 (47.4)	38 (100)	38 (100)	38 (100)	36 (94.7)	25 (65.8)
*P* value	**0.00**	**0.00**	**1.0**	**1.0**	**1.0**	**0.03**	**0.01**

Area blocking between the two groups was recorded. Axillary region dermatomal spread was significantly increased, but *T*_6_ and *T*_7_ dermatomal spread decreased in the TTP-PECS group. Data are expressed as mean ± SD (39 in the TTP-PECS group and 38 in the TPVB group)

*P < 0.01 or 0.05, versus the TPVB group

### Postoperative pain intensity

There were no significant differences in VAS scores at rest and during activity between the two groups at 2 h, 6 h, and 24 h postoperatively. At 12 h after the operation, the VAS scores at rest and during activity were both lower in the TTP-PECS group compared with the TPVB group (*P* = 0.00), as shown in Tables 4 and 5. However, in practice, this absolute difference of 0.5 at rest and 0.75 with activity may not be clinically significant. VAS scores at rest of all the patients were lower than 3, and VAS scores during activity of all the patients were lower than 5 suggesting none of the patients suffered severe pain in this clinical study. These results also imply our multi-mode pain managements for the participants were successful.

**Table 4:** Postoperative pain intensity at rest (VAS score at rest)

	TTP-PECS group	TPVB group	*P* value
Postoperative 2 h	1.25 ± 0.71	1.32 ± 0.68	**0.66**
Postoperative 6 h	1.70 ± 0.71	1.78 ± 0.68	**0.62**
Postoperative 12 h	2.11 ± 0.69*	2.60 ± 0.50	**0.00**
Postoperative 24 h	2.21 ± 0.48	2.29 ± 0.45	**0.45**

VAS scores at rest of all the patients were lower than 3, suggesting none of the patients suffered severe pain. There were no significant differences in VAS scores at rest between the two groups at 2 h, 6 h, and 24 h postoperatively. At 12 h postoperatively, the VAS scores at rest were lower in the TTP-PECS group compared with the TPVB group. Data are expressed as mean ± SD (39 in the TTP-PECS group and 38 in the TPVB group)

*P < 0.05, versus the TPVB group

**Table 5:** Postoperative pain intensity during activity (VAS score during activity)

	TTP-PECS group	TPVB group	*P* value
Postoperative 2 h	1.86 ± 0.68	1.81 ± 0.72	**0.76**
Postoperative 6 h	2.30 ± 0.73	2.27 ± 0.80	**0.86**
Postoperative 12 h	2.68 ± 0.68*	3.42 ± 0.57	**0.00**
Postoperative 24 h	3.12 ± 0.57	3.07 ± 0.62	**0.71**

VAS scores during the activity of all the patients were lower than 5, also suggesting none of the patients suffered severe pain. At 12 h after the operation, the VAS scores during the activity were lower in the TTP-PECS group compared with the TPVB group. Data are expressed as mean ± SD (39 in the TTP-PECS group and 38 in the TPVB group)

*P < 0.05, versus the TPVB group

### Pain-related mediators

To compare the pain relief of two nerve block methods in multiple directions, we detected the NPY, PGE2, and β-endorphin levels in the patient blood. The data are shown in Table 6.

**Table 6:** Effects of TTP-PECS and TPVB on pain-related mediators

Indicator	Group	Before surgery	Immediately after surgery	12 h after surgery	24 h after surgery
PGE_2_ (ng/l)	TTP-PECS group	28.60 ± 3.82	34.53 ± 5.69^#^	38.18 ± 6.25^#^*	42.38 ± 6.81^#^
	TPVB group	28.51 ± 3.81	34.80 ± 5.96^#^	46.66 ± 8.50^#^	42.66 ± 8.35^#^
	*P* value	**0.92**	**0.84**	**0.00**	**0.87**
NPY (μg/ml)	TTP-PECS group	79.01 ± 12.55	122.07 ± 15.74^#^	142.29 ± 22.93^#^*	149.11 ± 23.02^#^
	TPVB group	79.53 ± 12.39	122.67 ± 16.91^#^	160.22 ± 21.78^#^	148.13 ± 22.16^#^
	*P* value	**0.86**	**0.87**	**0.00**	**0.85**
β-Endorphin (ng/l)	TTP-PECS group	65.14 ± 6.69	69.64 ± 6.04^#^	74.37 ± 5.71^#^*	79.97 ± 5.67^#^
	TPVB group	64.36 ± 6.02	69.50 ± 6.72^#^	83.72 ± 8.17^#^	79.87 ± 7.76^#^
	*P* value	**0.59**	**0.92**	**0.00**	**0.95**

Compared with TPVB, TTP-PECS treatment significantly decreased the levels of pain-related mediators at 12 h after surgery. Data are expressed as mean ± SD (39 in the TTP-PECS group and 38 in the TPVB group)

^#^*P* < 0.05, versus before surgery

*P < 0.05, versus the TPVB group

In the two groups, compared with the levels before surgery, the NPY, PGE2, and β-endorphin increased after surgery (*P* < 0.05). However, their levels at 12 h postoperatively were all significantly lower in the TTP-PECS group than in the TPVB group (*P* < 0.05).

### Perioperative inflammatory response

To investigate the ability of TTP-PECS and TPVB to reduce the inflammatory response caused by the MRM surgery, we tested the expressions of the pro-inflammatory cytokines including IL-6, MCP-1, and TNF-α. The data are shown in Table 7.

**Table 7:** Effects of TTP-PECS and TPVB on pro-inflammatory cytokines

Indicator	Group	Before surgery	Immediately after surgery	12 h after surgery	24 h after surgery
IL-6 (pg/ml)	TTP-PECS group	36.22 ± 5.71	39.69 ± 4.92^#^	46.42 ± 5.38^#^*	42.45 ± 4.90^#^
	TPVB group	36.20 ± 6.10	39.21 ± 5.59^#^	49.37 ± 6.08^#^	42.92 ± 5.11^#^
	*P* value	**0.99**	**0.69**	**0.03**	**0.68**
MCP-1 (pg/ml)	TTP-PECS group	16.63 ± 1.58	20.85 ± 1.77^#^	36.28 ± 2.28^#^*	29.15 ± 2.36^#^
	TPVB group	17.06 ± 1.66	20.17± 2.02^#^	40.23 ± 2.86^#^	29.19 ± 2.41^#^
	*P* value	**0.25**	**0.12**	**0.00**	**0.94**
TNF-α (pg/ml)	TTP-PECS group	4.42 ± 0.73	8.06 ± 0.65^#^	13.59 ± 0.93^#^*	8.80 ± 0.79^#^
	TPVB group	4.48 ± 0.64	8.18 ±0.99^#^	16.14 ± 1.07^#^	8.82 ± 1.05^#^
	*P* value	**0.70**	**0.53**	**0.00**	**0.93**

Compared with TPVB, TTP-PECS treatment significantly decreased the levels of pro-inflammatory cytokines at 12 h after surgery. Data are expressed as mean ± SD (39 in the TTP-PECS group and 38 in the TPVB group)

^#^*P* < 0.05, versus before surgery

*P < 0.05, versus the TPVB group

In the two groups, compared with the level before surgery, IL-6, MCP-1, and TNF-α were overall upregulated immediately after surgery and at 12h and 24 h after surgery (*P* < 0.05), while the average levels of IL-6, MCP-1, and TNF-α at 12 h after surgery were all significantly lower in the TTP-PECS group than in the TPVB group (*P* < 0.05).

### Patients’ recovery quality

In order to evaluate the effect of improving the early recovery of patients in the two groups, we employed the QoR-40 and found that the global QoR-40 score was significantly higher in the TTP-PECS group than in the TPVB group. For the five dimensions of the QoR-40, the scores for support, pain, and emotional state were all significantly increased in patients receiving TTP-PECS on the postoperative days (*P* < 0.01), as shown in Table 8.

**Table 8:** The scores of QoR-40 on postoperative day

QoR-40 (score)	Physical comfort	Emotional state	Physical	Patient support	Pain	Total score
TTP-PECS group	53.38 ± 1.79	42.92 ± 0.77*	16.49 ± 1.10	31.64 ± 0.96*	31.69 ± 0.69*	176.13 ± 2.66*
TPVB group	53.08 ± 1.81	40.39 ± 1.48	16.34 ± 1.07	29.66 ± 1.12	29.26 ± 1.54	168.74 ± 3.42
*P* value	0.47	0.00	0.55	0.00	0.00	0.00

TTP-PECS treatment significantly increased the support, pain, and emotional state scores, as well as the total score after surgery. Data are expressed as mean ± SD (39 in the TTP-PECS group and 38 in the TPVB group)

*P < 0.01, versus the TPVB group

## Discussion

**Fig. 4:**
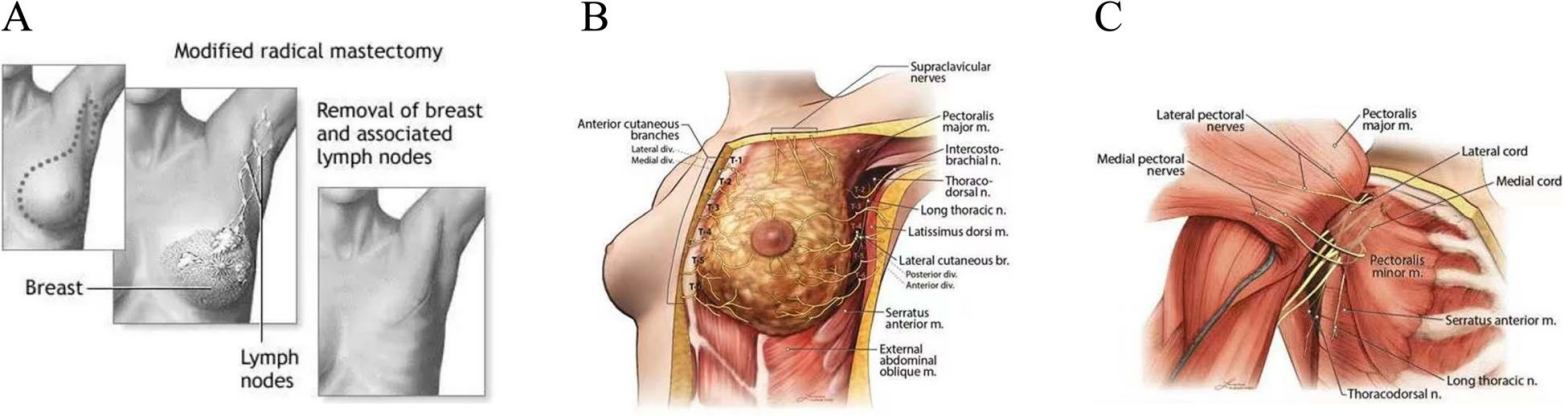
**A** Modified radical mastectomy. **B** Innervation of the breast. **C** Innervation of the thoracic

This present study demonstrated that TTP-PECS can provide better postoperative analgesia than TPVB in patients undergoing MRM. Eighty female breast cancer patients enrolled in this study, and all received standard unilateral MRM with sentinel lymph node dissection (SLND) and axillary dissection (ALND). A transverse or longitudinal fusiform incision was done on the surgical site, the breast tissues including the lesion were removed, and it was freed in the superficial layer of the pectoralis major fascia. After the lesion was removed, the axillary lymph nodes should be cleaned (Xie et al., 2022) (Fig. 4A).

From the anatomic point of view, the nerve supply to the breast is complex and innervated by a multiplicity of nerves. The anterolateral chest wall is innervated by the anterior cutaneous and lateral cutaneous branches of the *T*_2_–*T*_6_ nerves and long thoracic. Apart from the intercostal nerves, the lateral and the medial pectoral nerves also supply sensory and motor function to the skin and muscles of the chest wall. For procedures involving anterolateral, it is, therefore, necessary to block either the pertinent nerves at their origin or block their branches as they pierce their way into the subcutaneous tissue around the anterior axillary line or sternal border (Woodworth et al. 2017) (Fig. 4B, C).

Various regional anesthetic techniques have been used for pain management during breast surgery in recent years. TPVB has been widely studied for the prevention and treatment of acute pain and has been reported with varying degrees of success to provide analgesia after MRM (Simpson et al. 2014). However, TPVB have an inadequate block in the presence of axillary dissection, (Altıparmak et al. 2019). Patients undergoing TPVB frequently complain of postoperative pain in the axilla and upper limb due to sparing of the medial and lateral pectoral nerves (Mohamed et al., 2020). As we mentioned, MRM involves the removal of not only the breast but also the axillary lymph nodes. So, the main shortage of adequacy of TPVB is revealed during axillary dissection.

The pectoral nerve block (PECS I) aims to block targets medial and lateral pectoral nerves in the plane between the pectoralis major and pectoralis minor and block the lateral mammary area (Blanco 2011). It can provide analgesia related to surgical disruption of the pectoral muscles and related fascial structures. But PECS I block alone may lead to inadequate analgesia because of the highly innervated chest wall. Subsequently, Blanco et al. (2012) described a second version of the Pecs block called PECS II. The PECS II aims to block intercostal nerves 2 to 6, intercostobrachial, and the long thoracic nerves and produces a sensory loss in the axillary region in addition to the area affected by Pecs I block (Kelava et al. 2020). PECS II block incorporates analgesic blockade for axillary lymph node dissections, wide local excisions affecting the lateral breasts, and various breast reconstructive procedures (including breast expanders and subpectoral prosthesis insertion) (Maniker et al., 2020). However, intercostal nerve block has to be administered at multiple sites. Since PECS block can not provide adequate analgesia for procedures extending to the internal mammary area, Ueshima and Kitamura (2015) reported transversus thoracic muscle plane (TTP) block for analgesia of the inner breast region, and finally, TTP was added to suit the extent of surgery. Several studies (Zhang et al. 2018, You et al. 2019, Li et al., 2020) have confirmed this view. They found that TTP-PECS provided better analgesic efficacy for early postoperative analgesia than PECS II alone or PECS I combined with II in the patients undergoing MRM.

Based on these reports and the neural supply of the anterior chest wall and breast, we combined TTP with PECS together in this study and compare this TTP-PECS with the TPVB. We found that TTP-PECS shows consistent dermatomal spread in *T*_2_–*T*_6_ segments, even spread up to the *T*_7_ segment or more widely, as well as the axilla and upper limb areas. For TPVB, the sensory spread was usually observed at the level of injection (*T*_3_–*T*_7_), and less spread to *T*_2_ was observed, with very limited cephalad spread. TTP-PECS can complement the deficiency of TPVB. This suggested that both the two blocks can provide analgesia under the general anesthesia during the operation, but the blocking area of TTP-PECS was more suitable for the MRM.

However, we are more concerned with the postoperative analgesia, which is important for the early recovery and prognosis of patients. Firstly, we found that the duration of analgesia was significantly prolonged in patients receiving TTP-PECS as compared to the patients receiving TPVB. In the TTP-PECS group, the analgesia duration reached 12 h or more postoperatively.

Besides, in consideration of medical ethics, we did not want patients to suffer from moderate to severe pain after MRM. So, an intravenous PCIA pump with a background dose was used immediately after the operation in PACU, and flurbiprofen axetil was given as a remedial analgesia. VAS scores at rest of all the patients were lower than 3, and VAS scores during the activity of all the patients were lower than 5 suggesting none of the patients suffered severe pain in this clinical study. These results also imply our multi-mode pain managements for the participants were successful. There were no significant differences in VAS scores at rest and during activity between the two groups at 2 h, 6 h, and 24 h postoperatively, whereas at 12 h after the operation, the VAS scores at rest and during activity were both lower in the TTP-PECS group compared with the TPVB group. However, in practice, this absolute difference of 0.5 at rest and 0.75 with activity may not be clinically significant.

In addition, since the no difference in body weight between the two groups, the PCA press consumption (per bolus dosage multiply by the effective pressing times) could reflect the analgesic requirement. We observed marked advantages with the use of TTP-PECS. Total postoperative PCA fentanyl consumption, especially the PCA press consumption, and the rate of postoperative flurbiprofen axetil requirement were all decreased by the TTP-PECS compared with TPVB. These results suggested TTP-PECS to be more effective as a postoperative analgesic technique than TPVB which was consistent with several retrospective studies (Zhang et al. 2018, Li et al. 2021). Under real-time US guidance, with the deposition of local anesthetic drugs into the fascial planes, the TTP-PECS would be more accurate to provide higher analgesic efficacy for mastectomy and axillary clearance because of its complete paranesthesia of the hemithorax. Moreover, we also detected the NPY, PGE2, and β-endorphin in the patient blood and found that the serum levels of these pain-related mediators were also lower in the TTP-PECS group than those in the TPVB group at postoperative 12 h. These results indicate that TTP-PECS might be more effective in reducing hyperalgesia.

Also, we investigated the inflammatory response and found invasive MMR surgical procedures caused inflammatory response in varying degrees. However, the serum levels of these proinflammatory cytokines were all lower in the TTP-PECS group than those in the TPVB group at postoperative 12 h. Our finding is consistent with the previous report by Bagry et al. (2008). They reported a positive correlation between lower levels of inflammatory markers and pain in patients after knee surgery. Finally, we evaluated the early recovery of patients and found a remarkable improvement in life quality among patients treated with TTP-PECS. The improvement may be due to the fact that TTP-PECS relieved pain better.

Another important aspect is security. Actually, we discussed the security between these technical blocks in another study (Zhao et al. 2020). We found that TTP-PECS may reflect better mastering of the technique with time relative to the paravertebral technique. Also, TTP-PECS has a more stabled effect on perioperative hemodynamics. Furthermore, the incidence of complications such as spinal cord injury, epidural blockade, sympathectomy, and epidural hematoma was reduced in the TTP-PECS (Tighe and Karmakar, 2013). TTP-PECS is not restricted to the patients who are obese or use anticoagulants and also exhibited a satisfactory analgesic effect. It was also reported that most cases of TTP-PECS are performed under general anesthesia due to the advantage of easy positioning of the patient in the supine position. Comprehensive consideration of analgesic and security, TTP-PECS is an attractive alternative to TPVB for postoperative analgesia of MRM.

However, our study has several limitations. Firstly, a multicenter analysis was lacking. Secondly, we just comprehensively evaluated a series of short-term indicators; the postoperative pain outcomes and early recovery quality of patients were assessed only up to 24 h. Thirdly, on the basis of the available data from the current studies, we could not evaluate the efficacy in patients of different ages and for those with chronic pain. Clinical trials are needed to further explore and optimize this technique.

## Conclusion

TTP-PECS can provide better postoperative analgesia than TPVB; simultaneously, it has more advantages in reducing inflammatory response and promoting patient recovery. These results suggest that TTP-PECS is an attractive alternative to TPVB for patients undergoing MRM.

## Data Availability

All data generated or analyzed during this study are included in this published article. The data used to support the findings of this study and a complete and detailed test protocol can be obtained from the first author and corresponding author.

## Abbreviations

MRM: Modified radical mastectomy
TTP-PECS: Transversus thoracic muscle plane-pectoral nerve block
TPVB block: Thoracic paravertebral nerve block
QoR-40: Multidimensional Patient-Reported QoR-40 Questionnaire
US: Ultrasound
SLND: Sentinel lymph node dissection
ALND: Axillary dissection
BMI: Body mass index
ASA: American Society of Anesthesiologists
PACU: Post-anesthesia care unit
PCA: Patient-controlled analgesia
VAS: Visual analog scale
ELISA: Enzyme-linked immunosorbent assay
